# The Impact of Occupational Burnout on the Mental Health of Polish Nurses Working in Anesthesiology and Intensive Care Units

**DOI:** 10.3390/jcm15114294

**Published:** 2026-06-02

**Authors:** Beata Guzak, Aleksandra Łopatkiewicz, Iwona Kiersnowska, Edyta Krzych-Fałta

**Affiliations:** 1 Department of Nursing and Allied Health Professions, Centre of Postgraduate Medical Education, 01-813 Warsaw, Poland; beata.guzak@cmkp.edu.pl; 2 Department of Nursing Propaedeutics, Faculty of Health Sciences, Medical University of Warsaw, 01-828 Warsaw, Polandedyta.krzych-falta@wum.edu.pl (E.K.-F.)

**Keywords:** burnout syndrome, mental disorders, work–life balance

## Abstract

Background/Objectives Occupational burnout is a significant health concern among healthcare professionals, particularly among nurses working in anesthesiology and intensive care units who are exposed to high psychological workload. Previous studies indicate a relationship between burnout and deteriorating mental health; however, the role of individual burnout dimensions in shaping overall mental health remains insufficiently explored. Methods: This cross-sectional study included 842 nurses working in anesthesiology and intensive care units. Occupational burnout was assessed using the Oldenburg Burnout Inventory (OLBI), while mental health was evaluated using the General Health Questionnaire (GHQ-28). Sociodemographic variables and subjective assessment of work–life balance were also included. Statistical analyses comprised non-parametric tests, Spearman’s rank correlation, and multiple regression models. Results: The strongest correlations were observed between the exhaustion dimension and the overall GHQ-28 score (rS = 0.539; *p* < 0.001). Additionally, poorer work–life balance was associated with increased symptoms of mental health disorders (rS = 0.42; *p* < 0.001). Regression analysis showed that gender, exhaustion, and work–life balance were significant predictors of mental health (*p* < 0.001). Conclusions: The results indicate a significant relationship between occupational burnout and mental health in nurses working in anesthesiology and intensive care units. In particular, exhaustion and work–life balance were identified as important predictors of mental health outcomes.

## 1. Introduction

Occupational burnout is one of the most significant challenges in contemporary occupational medicine and public health, particularly among individuals employed in the healthcare system. The occurrence of burnout, combined with prolonged exposure to work-related stress, high clinical responsibility, time pressure, and exposure to patient suffering, significantly increases the risk of mental health disorders in this occupational group [1]. This phenomenon has not only an individual dimension but also a systemic one, affecting the quality of patient care, clinical safety, and the stability of the healthcare workforce [2]. The importance of mental health in the workplace has also been emphasized by the World Health Organization (WHO), which indicates that unfavorable organizational and psychosocial working conditions constitute a significant risk factor for the deterioration of mental health among healthcare workers [3]. Recent reports also highlight that protecting the mental health of healthcare professionals is one of the key priorities of modern public health [4].

Occupational burnout is defined as a syndrome resulting from chronic work-related stress, with exhaustion and disengagement from professional duties as its central components [5]. It is increasingly recognized that burnout is associated with significant clinical consequences, including impairments in cognitive and emotional performance as well as a deterioration of overall mental health among healthcare workers [6]. Among medical professions, nurses working in anesthesiology and intensive care units represent a particularly vulnerable group. Their work takes place in a high-risk clinical environment and involves a demand for rapid decision-making, constant vigilance, and intense psychological and physical workload [7,8]. Working in operating rooms and intensive care settings exacerbates stress and increases susceptibility to occupational burnout as well as mental health disorders [9,10]. The experiences of the COVID-19 pandemic have further highlighted the scale of systemic burdens and increased pressure on this professional group [11]. Studies conducted among nurses working in anesthesiology and intensive care units indicate that high levels of occupational stress are associated with impaired psychological performance and, consequently, a reduced quality of life, with psychosocial factors playing a significant role in moderating these relationships [12].

Studies conducted in Poland indicate that nurses experience high levels of occupational stress and burnout, particularly in hospital settings. Previous research has demonstrated moderate to high levels of burnout and significant associations between occupational stress and burnout among Polish nurses [13].

However, existing studies have primarily focused on selected aspects of burnout or its determinants, without a comprehensive assessment of overall mental health using standardized psychometric tools. Furthermore, analyses of the Polish literature suggest the presence of gaps in empirical research, with limited sample sizes and a lack of integrated approaches to assessing burnout and broader psychological outcomes [14]. Despite the growing body of research on occupational burnout among nurses, there remains a lack of studies that simultaneously assess burnout and general mental health using validated instruments such as the Oldenburg Burnout Inventory (OLBI) and the General Health Questionnaire (GHQ-28), particularly among nurses working in anesthesiology and intensive care units in Poland.

Identifying burnout as a predictor of deteriorating mental health underscores the importance of integrating burnout assessment into routine occupational health evaluations. This may also provide a basis for developing preventive and intervention strategies in line with World Health Organization recommendations on workplace mental health promotion and the evidence supporting the effectiveness of systemic interventions in reducing burnout [3,15].

In recent years, increasing attention has also been paid to the importance of work–life balance as a key factor influencing the mental health of healthcare workers. Disruption of this balance is associated with increased stress levels and a higher risk of occupational burnout [15,16].

Therefore, there is a need for comprehensive research examining the relationship between occupational burnout and overall mental health among nurses working in high-risk clinical environments.

The aim of this study was to assess the relationship between occupational burnout and general mental health using validated instruments (OLBI and GHQ-28) among nurses working in anesthesiology and intensive care units in Poland.

## 2. Materials and Methods

The study included nurses working professionally in the field of anesthesiology and intensive care, currently employed in clinical settings. Participants were recruited using a convenience sampling method.

The exclusion criteria were: employment outside the field of anesthesiology and intensive care (e.g., working exclusively at a university or outside the healthcare system).

The study was conducted in March 2024 among individuals who had completed specialization training in anesthesiology and intensive care. A total of 864 questionnaires were collected; 22 were excluded due to missing data, incorrectly reported age or a primary workplace unrelated to anesthesiology or intensive care (e.g., a university). Ultimately, data from 842 participants (completed questionnaires) were included in the analysis.

The sample size was calculated based on the number of nurses holding a specialization in anesthesiology and intensive care in Poland (19,541 in 2025). Assuming a confidence level of 98%, a maximum error of 4%, and a fraction size of 0.5, the sample size amounted to 811 nurses [17].

The mean age of the respondents was 34.9 ± 9.5 years (Mdn = 31; range: 25–61), and the mean length of service was 10.7 ± 9.4 years (Mdn = 6; range: 2–38). Table 1 presents the detailed characteristics of the study group.

### Research Instruments

The level of occupational burnout was assessed using the Polish adaptation [18] of Oldenburg Burnout Inventory (OLBI), which measures two core dimensions of burnout: exhaustion and disengagement from work [19, 20]. The OLBI demonstrated good reliability (α = 0.83). Analysis of its subscales indicated that both the disengagement scale (α = 0.71) and the exhaustion scale (α = 0.74) achieved acceptable values, meeting reliability criteria for scientific research. The OLBI allows for the assessment of both affective and cognitive aspects of burnout, which increases its suitability in studies involving healthcare professionals [19, 20].

General mental health status was assessed using the Polish adaptation [21] of the General Health Questionnaire (GHQ-28) [22], which enables the evaluation of four domains: somatic symptoms, anxiety and insomnia symptoms, social dysfunction, and depressive symptoms. Higher GHQ-28 scores indicate poorer mental health. The GHQ demonstrated very high reliability (α = 0.93), indicating excellent internal consistency. Similarly high Cronbach’s alpha values were obtained for the subscales: Severe Depression (α = 0.91), Anxiety and Insomnia (α = 0.88), and Social Dysfunction (α = 0.85), confirming good reliability. The Somatic Symptoms subscale reached α = 0.83, also within the range of high reliability. The GHQ-28 allows for the identification of somatic, anxiety-related, social dysfunctioning, and depressive symptoms, providing a comprehensive assessment of an individual’s mental health [22].

The questionnaire also included items concerning sociodemographic data, professional experience (length of service), and work–life balance among the surveyed nurses. Subjective assessment of work–life balance was measured using a single-item question with a four-point response scale (1 = “definitely yes”, 2 = “rather yes”, 3 = “rather no”, 4 = “definitely no”).

The study was conducted in accordance with the principles of the Declaration of Helsinki. Approval was obtained from the Bioethics Committee of the Medical University of Warsaw (approval no.: AKBE/305/2023). Participation in the study was voluntary and anonymous, and respondents were informed about the purpose of the study and their right to withdraw at any stage.

The normality of the distribution was assessed using the Shapiro–Wilk test. Due to the lack of normal distribution, non-parametric tests were applied. Quantitative variables were analyzed using the Mann–Whitney U test and the Kruskal–Wallis ANOVA. Correlations were assessed using Spearman’s rank correlation coefficient. The level of statistical significance was set at *p* < 0.05.

Multiple regression models were constructed using the following independent variables: length of service, gender, work–life balance, and OLBI subscales (disengagement and exhaustion). The GHQ total score and its subscales (Somatic Symptoms, Anxiety and Insomnia, Social Dysfunction, Severe Depression) were used as dependent variables.

## 3. Results

The distribution of GHQ-28 and OLBI scores in the study group of nurses is presented in Table 2.

Correlation analysis revealed significant positive interdependence between the level of occupational burnout assessed with the OLBI and overall mental health measured by the GHQ. The strongest associations were observed between the total GHQ score and the OLBI exhaustion subscale (*rho* = 0.539; *p* < 0.001), as well as the total OLBI score (*rho* = 0.491; *p* < 0.001). Compared with disengagement, exhaustion showed stronger correlations with all GHQ subscales (Table 3).

Statistically significant differences between women and men were found in the Somatic Symptoms, Anxiety and Insomnia, and Social Dysfunction subscales, as well as in the total GHQ score and the OLBI exhaustion scale, with women obtaining higher scores (*p* < 0.018). No significant differences were observed in the Severe Depression subscale, the disengagement scale, or the total OLBI score (*p* > 0.05) (Table 4).

A significant, progressive increase in the severity of occupational burnout and mental health disorder symptoms was observed as the subjective assessment of work–life balance deteriorated (*p* < 0.001; Table 5).

### Predictors of Mental Health—Regression Analysis

All constructed models were statistically significant (*p* < 0.001) and indicated a strong influence of variables such as gender and work–life balance on GHQ scores. In terms of occupational burnout measured with the OLBI, the exhaustion dimension had the strongest impact on mental health, significantly increasing the severity of mental health disorder symptoms across all analyzed GHQ domains (*p* < 0.001).

Subjective assessment of the ability to maintain work–life balance was a significant predictor in all models (*p* ≤ 0.004). Gender was also a significant predictor for most analyzed dimensions of mental health, with the exception of Severe Depression (*p* = 0.622). In contrast, disengagement had limited predictive value and reached statistical significance only in the Severe Depression model (*p* = 0.008) (Table 6).

Table 6 presents the results of the regression analysis. Exhaustion and work–life balance were identified as significant predictors of mental health (*p* < 0.001), with exhaustion showing the strongest effect.

## 4. Discussion

The present study confirms a significant correlation between occupational burnout and deterioration of overall mental health among nurses working in anesthesiology and intensive care units. The obtained results indicate that among the analyzed dimensions of burnout, exhaustion plays a key role, emerging as the strongest and most stable predictor of the severity of psychological symptoms measured with the GHQ-28. This relationship was observed both for the overall GHQ score and all its subscales (*p* < 0.001).

The findings are consistent with previous studies demonstrating a strong association between occupational burnout and poorer mental health among nursing staff [1,6]. In contrast to many earlier analyses that focused on individual psychological symptoms or used different measurement tools, the present study enables a comprehensive assessment of mental health using the GHQ-28 as an outcome variable. This approach allows for a more thorough clinical interpretation of the consequences of occupational burnout, encompassing somatic, emotional, and social dimensions of functioning of nurses examined. Unlike previous studies that primarily focused on burnout or selected psychological symptoms [6,10,23], the present findings provide new data on the simultaneous assessment of occupational burnout and overall mental health using the OLBI and GHQ-28 instruments.

According to meta-analyses conducted by Woo et al. and Gómez-Urquiza et al., nurses working in environments with high emotional and organizational demands are at particularly high risk of burnout and associated mental health disorders. Aforementioned authors indicate that fields such as anesthesiology, intensive care, and emergency medicine are among the most psychologically demanding nursing specializations [10,23], which may explain the high severity of exhaustion and its strong association with GHQ-28 symptoms observed in the studied population.

It is noteworthy that in the present study, exhaustion, rather than disengagement, proved to be a significantly stronger predictor of mental health, which is consistent with the conceptualization of burnout as a process progressing from energy depletion to subsequent disengagement from work. Thus, exhaustion appears to be the burnout dimension with direct clinical relevance, while disengagement may act as a secondary adaptive response to chronic overload. Similar observations have been reported by Demerouti et al. and Halbesleben and Demerouti, who indicated that the exhaustion component most directly translates into clinically significant psychological outcomes [18,19].

Comparable correlations have also been observed in other high-stress nursing specializations. A multicenter study conducted among psychiatric nurses in six European countries demonstrated that emotional exhaustion was the strongest predictor of deteriorating mental health measured with the GHQ-28, regardless of age and length of service. Those findings confirm that exhaustion plays a central role in shaping the mental health of nurses working under conditions of prolonged and intensive occupational stress [24].

The higher severity of psychological symptoms observed among women is consistent with population-based studies indicating significant gender differences in psychological distress among working individuals [25]. Those findings are also in line with the meta-analysis by Purvanova and Muros, which showed that women report higher levels of emotional exhaustion, a key component of occupational burnout [26]. Similar patterns have been observed among healthcare workers, where women exhibited higher levels of depressive symptoms, anxiety, and stress compared to men [27]. This phenomenon may result from differences in emotional reactivity and coping strategies, as well as a greater psychosocial burden associated with combining professional and family roles. In the context of nursing, which inherently involves high emotional demands, those factors may further increase vulnerability to mental health deterioration.

Additionally, the significant role of the subjective assessment of work–life balance confirms the influence of organizational and psychosocial factors on the development of mental health disorders. Previous studies have shown that lack of work–life balance significantly reduces psychological wellbeing among healthcare workers, regardless of demographic characteristics or length of service [15,16,28,29].

The results of the present study are consistent with those observations, indicating that perceived difficulty in maintaining work–life balance is associated with increased severity of psychological symptoms measured by the GHQ-28. This correlation can be interpreted in light of the Job Demands–Resources (JD-R) model, according to which excessive job demands and work–life conflict lead to increased psychological stress and exhaustion. Similar mechanisms have been described by Abdou et al. and Farivar et al., who demonstrated that work–life conflict and high job demands are associated with higher severity of psychological distress, regardless of occupational context [30,31].

The obtained results are consistent with studies indicating the significant role of psychological resources in mitigating the negative effects of occupational stress among nurses working in anesthesiology and intensive care units. A study by Peñacoba et al. showed that higher levels of resilience and self-efficacy are associated with better quality of life and lower stress levels in this group. Peñacoba et al. explain that those resources may act as protective buffers against chronic occupational strain, which aligns with the present findings, emphasizing the key role of exhaustion and organizational factors in shaping mental health [32].

Importantly, the significance of work–life balance persisted even after controlling for burnout levels in regression models, suggesting its independent impact on nurses’ mental health. Therefore, work–life imbalance should not be viewed solely as a consequence of burnout but also as a distinct mechanism affecting psychological functioning. From a clinical perspective, this implies that interventions focused exclusively on reducing burnout symptoms may be insufficient if they do not address structural and organizational working conditions. The results suggest that both occupational exhaustion and work–life balance are important factors associated with mental health among nurses working in anesthesiology and intensive care units. It is worth emphasizing that both exhaustion and impaired work–life balance remained significant predictors of mental health, although exhaustion demonstrated a particularly strong and consistent effect across the analyzed models.

Moreover, it was shown that subjective assessment of disrupted work–life balance was significantly associated with poorer mental health. This correlation is also supported by studies conducted in general populations, which indicate that work–life imbalance is associated with lower life satisfaction and poorer mental health [33]. These findings highlight the importance of organizational and psychosocial factors as key determinants of mental wellbeing among nursing staff. The observed correlations suggest that both occupational exhaustion and lack of work–life balance constitute significant risk factors for mental health deterioration.

The obtained results carry important practical and clinical implications for the mental health care of nurses working in high-demand settings such as anesthesiology and intensive care. Identifying occupational exhaustion as the strongest and most stable predictor of deteriorating mental health indicates that its systematic assessment should be an integral component of preventive strategies in occupational medicine.

The use of tools such as the OLBI and GHQ-28 enables early detection of both occupational burnout and clinically relevant symptoms of mental health disorders, allowing for the implementation of targeted preventive interventions before the development of full-blown mental disorders.

Furthermore, the research significance of impaired work–life balance in the deterioration of mental health highlights the need to implement organizational solutions aimed at optimizing working hours and shift work, as well as ensuring access to psychological support. The study findings indicate that effective prevention of mental health disorders among nurses should combine individual-level interventions with systemic actions that take into account working conditions and organizational factors.

## 5. Limitations

This study has several limitations that should be considered when interpreting the results. First, the cross-sectional design does not allow for establishing causal relationships between occupational burnout and mental health. Second, the data were collected using self-report questionnaires, which may be associated with response bias and subjective interpretation of the questions. Third, the study was conducted among nurses working in anesthesiology and intensive care units in Poland, which may limit the generalizability of the findings to other professional groups or healthcare systems. Additionally, some potentially relevant variables, such as the number of night shifts, detailed job characteristics, or substance use, were not included in the analysis, which may influence the observed relationships.

## 6. Conclusions

The results of this study indicate a significant relationship between occupational burnout and mental health among nurses working in anesthesiology and intensive care units. In particular, exhaustion and work–life balance were identified as important predictors of mental health outcomes. These findings contribute to a better understanding of the factors associated with mental health in this occupational group and may provide a basis for future research and the development of preventive strategies.

## Figures and Tables

**Table 1:** Characteristics of the study group (*n* = 842).

	*n*	%
gender		
female	751	89.19
male	91	10.81
marital status		
single	188	22.33
married/in a relationship	585	69.48
divorced/separated	60	7.13
widowed	9	1.07
education		
secondary vocational	39	4.63
bachelor’s degree	139	16.51
master’s degree	664	78.86

**Table 2:** GHQ and OLBI scale results in the group of nurse anesthetists (*n* = 842).

	Mean (SD)	Median (Min–Max)
GHQ	55.94 ± 12.26	54 (28–106)
Somatic Symptoms	15.4 ± 4.05	15 (7–28)
Anxiety and Insomnia	15.91 ± 4.52	15 (7–28)
Social Dysfunction	14.66 ± 2.74	14 (7–28)
Severe Depression	9.97 ± 3.91	8 (7–28)
OLBI	39.26 ± 6.59	39 (18–61)
Disengagement scale	19.04 ± 3.6	19 (9–32)
Exhaustion scale	20.22 ± 3.61	20 (8–31)

**Table 3:** Correlation between GHQ and OLBI scales (*n* = 842).

	RSpearman	*p*
GHQ score		
OLBI score	0.491201	<0.001
Disengagement scale	0.366514	<0.001
Exhaustion scale	0.538845	<0.001
Somatic Symptoms		
OLBI score	0.435534	<0.001
Disengagement scale	0.332312	<0.001
Exhaustion scale	0.470029	<0.001
Anxiety and Insomnia		
OLBI score	0.400262	<0.001
Disengagement scale	0.272477	<0.001
Exhaustion scale	0.464193	<0.001
Social Dysfunction		
OLBI suma	0.415179	<0.001
Disengagement scale	0.328647	<0.001
Exhaustion scale	0.437486	<0.001
Severe Depression		
OLBI score	0.352610	<0.001
Disengagement scale	0.293279	<0.001
Exhaustion scale	0.361277	<0.001

**Table 4:** Differences between groups (*n* = 842).

	Female (*n* = 751)		Male (*n* = 91)		*Z*	*p*
	Mean (SD)	Median (Min–Max)	Mean (SD)	Median (Min–Max)		
GHQ	56.4 ± 12.2	55 (28–106)	52.14 ± 12.19	50 (30–90)	3.34	0.001
Somatic Symptoms	15.59 ± 4	15 (7–28)	13.87 ± 4.18	13 (7–27)	4.30	<0.001
Anxiety and Insomnia	16.11 ± 4.49	15 (7–28)	14.27 ± 4.38	14 (7–26)	3.64	<0.001
Social Dysfunction	14.75 ± 2.72	14 (7–28)	13.87 ± 2.77	14 (7–22)	2.37	0.018
Severe Depression	9.95 ± 3.84	8 (7–28)	10.13 ± 4.49	8 (7–27)	0.86	0.391
OLBI	39.31 ± 6.41	39 (18–60)	38.87 ± 7.96	38 (21–61)	1.21	0.225
Disengagement scale	18.99 ± 3.48	19 (9–31)	19.44 ± 4.49	19 (10–32)	−0.21	0.835
Exhaustion scale	20.32 ± 3.55	20 (8–31)	19.43 ± 4.03	19 (11–29)	2.25	0.025

**Table 5:** Perceived ability to maintain work–life balance (*n* = 842).

	I Definitely Yes (*n* = 91)	II Rather Yes (*n* = 540)	III Rather No (*n* = 179)	IV Definitely No (*n* = 32)	*H*	*p*
	Median (Min–Max)	Median (Min–Max)	Median (Min–Max)	Median (Min–Max)		
GHQ	48 (32–93)	53 (28–105)	59 (31–99)	67 (43–106)	75.22	<0.001
I ≠ II (*p* = 0.007) I ≠ III, I ≠ IV, (*p* < 0.001) II ≠ III, II ≠ IV (*p* < 0.001)						
Somatic Symptoms	13 (7–26)	14 (7–28)	17 (8–26)	19 (10–27)	71.49	<0.001
I ≠ III, I ≠ IV, (*p* < 0.001) II ≠ III, II ≠ IV (*p* < 0.001)						
Anxiety and Insomnia	14 (7–28)	15 (7–28)	17 (7–27)	19 (11–28)	59.20	<0.001
I ≠ II (*p* = 0.009) I ≠ III, I ≠ IV, (*p* < 0.001) II ≠ III, II ≠ IV (*p* < 0.001)						
Social Dysfunction	14 (7–21)	14 (7–25)	14 (7–23)	15 (7–28)	38.06	<0.001
I ≠ III, I ≠ IV, (*p* < 0.001) II ≠ III (*p* = 0.001)						
Severe Depression	7 (7–21)	8 (7–27)	10 (7–28)	9 (7–25)	32.31	<0.001
I ≠ III, (*p* < 0.001) II ≠ III, (*p* < 0.001)						
OLBI	35 (20–57)	38 (20–60)	43 (18–55)	44.5 (23–61)	86.03	<0.001
I ≠ II, I ≠ III, I ≠ IV, (*p* < 0.001) II ≠ III, II ≠ IV (*p* < 0.001)						
Disengagement scale	17 (9–31)	19 (10–31)	20 (9–29)	22 (13–32)	67.41	<0.001
I ≠ II (*p* = 0.006) I ≠ III, I ≠ IV, (*p* < 0.001) II ≠ III, II ≠ IV (*p* < 0.001)						
Exhaustion scale	18 (10–29)	20 (9–29)	22 (9–31)	22.5 (8–29)	73.45	<0.001
I ≠ II (*p* = 0.001) I ≠ III, I ≠ IV, (*p* < 0.001) II ≠ III, II ≠ IV (*p* < 0.001)						

**Table 6:** Predictors of mental health (GHQ-28)—multiple regression analysis adjusted for length of service (*n* = 842).

Variable	*b*	Standard Error	*t*	95% Confidence Interval		*p*
Model 1: GHQ (total)						
*p* < 0.001 *, R-squared = 0.284, MSE = 90.353						
Gender	−3.29	1.18	−2.79	(−5.60	−0.97)	0.005 *
Work-life balance	3.14	0.57	5.50	(2.02	4.27)	<0.001 *
Disengagement scale	0.08	0.14	0.56	(−0.19	0.34)	0.578
Exhaustion scale	1.46	0.14	10.69	(1.19	1.73)	<0.001 *
Model 2: Somatic Symptoms						
*p <* 0.001 *, R-squared = 0.242, MSE = 10.439						
Gender	−1.41	0.40	−3.52	(−2.20	−0.62)	<0.001 *
Work-life balance	1.04	0.19	5.34	(0.66	1.42)	<0.001 *
Disengagement scale	0.01	0.05	0.30	(−0.08	0.10)	0.767
Exhaustion scale	0.44	0.05	9.37	(0.34	0.53)	<0.001 *
Model 3: Anxiety and Insomnia						
*p* < 0.001 *, R-squared = 0.237, MSE = 13.041						
Gender	−1.40	0.45	−3.13	(−2.28	−0.52)	0.002 *
Work-life balance	1.09	0.22	5.00	(0.66	1.51)	<0.001 *
Disengagement scale	−0.10	0.05	−1.94	(−0.20	0.00)	0.052
Exhaustion scale	0.57	0.05	10.88	(0.46	0.67)	<0.001 *
Model 4: Social Dysfunction						
*p* < 0.001 *, R-squared = 0.192, MSE = 0.354						
Gender	−0.68	0.28	−2.45	(−1.23	−0.14)	0.015 *
Work-life balance	0.43	0.14	3.15	(0.16	0.69)	0.002 *
Disengagement scale	0.03	0.03	1.04	(−0.03	0.10)	0.299
Exhaustion scale	0.27	0.03	8.25	(0.20	0.33)	<0.001 *
Model 5: Severe Depression						
*p* < 0.001 *, R-squared = 0.103, MSE = 11.516						
Gender	0.21	0.42	0.49	(−0.62	1.03)	0.622
Work-life balance	0.59	0.20	2.90	(0.19	0.99)	0.004 *
Disengagement scale	0.13	0.05	2.65	(0.03	0.23)	0.008 *
Exhaustion scale	0.19	0.05	3.97	(0.10	0.29)	<0.001 *

* *p* < 0.05.

## Data Availability

The data presented in this study are available upon reasonable request from the corresponding author. The data are not publicly available due to privacy and ethical restrictions.

## Abbreviations

The following abbreviations are used in this manuscript:

OLBI	Oldenburg Burnout Inventory
GHQ-28	General Health Questionnaire (28-item version)
rS	Spearman’s rank correlation coefficient
*p*	*p*-value

## References

[1] Maslach C., Leiter M.P. (2016). Understanding the burnout experience: Recent research and its implications for psychiatry. World Psychiatry.

[2] Dyrbye L.N., Shanafelt T.D. (2016). A narrative review on burnout experienced by medical students and residents. Med. Educ..

[3] World Health Organization (2022). Mental Health at Work: Policy Brief.

[4] Chirico F., Ferrari G., Nucera G., Szarpak L., Crescenzo P., Ilesanmi O. (2021). Prevalence of anxiety, depression, burnout syndrome, and mental health disorders among healthcare workers during the COVID-19 pandemic: A rapid umbrella review. J. Health Soc. Sci..

[5] Schaufeli W.B., De Witte H., Desart S. (2020). Manual Burnout Assessment Tool (BAT)—Version 2.0.

[6] Salvagioni D.A.J., Melanda F.N., Mesas A.E., González A.D., Gabani F.L., Andrade S.M. (2017). Physical, psychological and occupational consequences of job burnout: A systematic review. PLoS ONE.

[7] Gillespie M., Melby V. (2003). Burnout among nursing staff in accident and emergency and acute medicine: A comparative study. J. Clin. Nurs..

[8] Adriaenssens J., De Gucht V., Maes S. (2015). Determinants and prevalence of burnout in emergency nurses: A systematic review. J. Adv. Nurs..

[9] Mealer M., Burnham E.L., Goode C.J., Rothbaum B., Moss M. (2009). The prevalence and impact of post-traumatic stress disorder and burnout syndrome in nurses. Depress. Anxiety.

[10] Woo T., Ho R., Tang A., Tam W. (2020). Global prevalence of burnout symptoms among nurses: A meta-analysis. J. Psychiatr. Res..

[11] Azoulay E., De Waele J., Ferrer R., Staudinger T., Borkowska M., Povoa P., Iliopoulou K., Artigas A., Schaller S.J., Hari M.S. (2020). Symptoms of burnout in intensive care unit specialists facing the COVID-19 outbreak. Ann. Intensive Care.

[12] Peñacoba C., Catalá P., Velasco L., Carmona-Monge F.J., García-Hedrera F.J., Gil-Almagro F. (2021). Stress and quality of life of intensive care nurses during the COVID-19 pandemic: Self-efficacy and resilience as resources. Nurs. Crit. Care.

[13] Tomaszewska K., Kowalczuk K., Kadučáková H., Lehotská M., Papp K., Majchrowicz B. (2024). Occurrence of Stress and Burnout Among Nurses Employed in a Psychiatric Hospital and a Somatic Hospital—A Comparative Analysis (Nursing Workload KEGA č. 011KU-4/2024). Healthcare.

[14] Kołodziej K., Wilczek-Rużyczka E., Majda A. (2025). The Relationship Between Sense of Coherence and Occupational Burnout Among Psychiatric Nurses: A Cross-Sectional Study in Inpatient Psychiatric Wards in Poland. Nurs. Rep..

[15] West C.P., Dyrbye L.N., Erwin P.J., Shanafelt T.D. (2016). Interventions to prevent and reduce physician burnout: A systematic review and meta-analysis. Lancet.

[16] Mudallal R.H., Othman W.A.M., Al Hassan N.F. (2017). Nurses’ burnout: The influence of leader empowering behaviors. Inquiry.

[17] Naczelna Izba Pielęgniarek i Położnych (2025). Specjalizacje Pielęgniarek i Położnych w Polsce. https://nipip.pl/wp-content/uploads/2026/01/05-2025-12-31-Specjalizacje.pdf.

[18] Gruszczyńska E., Basińska B.A. (2015). Polish adaptation of the Oldenburg Burnout Inventory (OLBI). Pol. Psychol. Bull..

[19] Demerouti E., Bakker A.B., Vardakou I., Kantas A. (2003). The convergent validity of two burnout instruments: A multitrait–multimethod analysis. Eur. J. Psychol. Assess..

[20] Halbesleben J.R.B., Demerouti E. (2005). The construct validity of an alternative measure of burnout. Work Stress.

[21] Makowska Z., Merecz D. (2001). Polska Adaptacja Kwestionariuszy GHQ-12 i GHQ-28.

[22] Goldberg D.P., Hillier V.F. (1979). A scaled version of the General Health Questionnaire. Psychol. Med..

[23] Gómez-Urquiza J.L., De la Fuente-Solana E.I., Albendín-García L., Vargas-Pecino C., Ortega-Campos E., Cañadas-De la Fuente G.A. (2017). Prevalence of burnout syndrome in emergency nurses: A meta-analysis. Crit. Care Nurse.

[24] Łopatkiewicz A., Kwaśnicka A., Nowicki P., Furmańczyk K., Zieliński W., Woynarowska M., Krzych-Fałta E. (2023). Occupational burnout and mental health. Adv. Cogn. Psychol..

[25] Viertiö S., Kiviruusu O., Piirtola M., Kaprio J., Korhonen T., Marttunen M., Suvisaari J. (2021). Psychological distress in the working population. BMC Public Health.

[26] Purvanova R.K., Muros J.P. (2010). Gender differences in burnout: A meta-analysis. J. Vocat. Behav..

[27] Lai J., Ma S., Wang Y., Cai Z., Hu J., Wei N., Wu J., Du H., Chen T., Li R. (2020). Factors associated with mental health outcomes among healthcare workers exposed to COVID-19. JAMA Netw. Open.

[28] Allen T.D., Herst D.E.L., Bruck C.S., Sutton M. (2000). Consequences associated with work-to-family conflict. J. Occup. Health Psychol..

[29] Søvold L.E., Naslund J.A., Kousoulis A.A., Saxena S., Qoronfleh M.W., Grobler C., Münter L. (2021). Mental health of healthcare workers as a global priority. Front. Public Health.

[30] Abdou A.H., El-Amin M.A.-M.M., Mohammed E.F.A., Alboray H.M.M., Refai A.M.S., Almakhayitah M.Y., Albohnayh A.S.M., Alismail A.M., Almulla M.O., Alsaqer J.S. (2024). Work stress and psychological distress. Front. Psychol..

[31] Farivar F., Geneste L., Esmaeelinezhad O., Yaghoubi M. (2025). Work-Life Conflict and Work Engagement: Which Drives the Other? A Fuzzy-Set Approach. Hum. Resour. Dev. Int..

[32] Peñacoba C., Velasco L., Catalá P., Gil-Almagro F., García-Hedrera F.J., Carmona-Monge F.J. (2021). Resilience and anxiety among ICU professionals. Nurs. Crit. Care.

[33] Haar J.M., Russo M., Suñe A., Ollier-Malaterre A. (2014). Outcomes of work–life balance. J. Vocat. Behav..

